# Miz1 Is a Critical Repressor of *cdkn1a* during Skin Tumorigenesis

**DOI:** 10.1371/journal.pone.0034885

**Published:** 2012-04-11

**Authors:** Jan Hönnemann, Adrián Sanz-Moreno, Elmar Wolf, Martin Eilers, Hans-Peter Elsässer

**Affiliations:** 1 Department of Cytobiology, Philipps-University Marburg, Germany; 2 Theodor-Boveri-Institute, Biocentre, University of Würzburg, Germany; University of Saarland Medical School, Germany

## Abstract

The transcription factor Miz1 forms repressive DNA-binding complexes with the Myc, Gfi-1 and Bcl-6 oncoproteins. Known target genes of these complexes encode the cyclin-dependent kinase inhibitors (CKIs) *cdkn2b* (p15^Ink4^), *cdkn1a* (p21^Cip1^), and *cdkn1c* (p57^Kip2^). Whether Miz1-mediated repression is important for control of cell proliferation *in vivo* and for tumor formation is unknown. Here we show that deletion of the Miz1 POZ domain, which is critical for Miz1 function, restrains the development of skin tumors in a model of chemically-induced, Ras-dependent tumorigenesis. While the stem cell compartment appears unaffected, interfollicular keratinocytes lacking functional Miz1 exhibit a reduced proliferation and an accelerated differentiation of the epidermis in response to the tumor promoter 12-O-tetradecanoylphorbol-13-acetate (TPA). Tumorigenesis, proliferation and normal differentiation are restored in animals lacking *cdkn1a*, but not in those lacking *cdkn2b*. Our data demonstrate that Miz1-mediated attenuation of cell cycle arrest pathways via repression of *cdkn1a* has a critical role during tumorigenesis in the skin.

## Introduction

Miz1 (*M*yc-*i*nteracting *z*inc finger protein 1; Zbtb17) is a zinc finger transcription factor that forms a complex with the Myc oncoprotein [1]. In addition to 13 zinc fingers that are clustered in the central and C-terminal part of the protein, Miz1 bears a POZ (*po*xvirus *z*inc finger protein) domain at the N-terminus [2]. POZ domains are found in a variety of different transcription factors and can confer hetero- or homodimerization as well as tetramerisation [3], [4]. The POZ domain of Miz1 forms tetramers [3] and is required for a stable association of Miz1 with chromatin [5], [6]. In addition, Miz1 lacking the POZ domain can also interact with other proteins. As a result, the POZ domain is required both for transcriptional activation and repression by Miz1.

Among the proteins which interact with Miz1 independent of the POZ domain is Myc, which binds to Miz1 between the zinc finger domains 12 and 13 [1]. This complex represses the transcription of genes including *cdkn2b* (encoding p15^Ink4b^), *cdkn1a* (encoding p21^Cip1^), *cdkn1c* (encoding p57^Kip2^) and *mxd4* (encoding Mad4) [7]–[10]. In the absence of Myc, Miz1 activates transcription of a number of genes including Bcl-2 [11], clusterin, several integrins and other proteins involved in cell adhesion [12], in a concerted manner with other transcription factors. For example, Miz1 synergizes with the Smad complex during the TGF-ß mediated activation of *cdkn2b* expression [7], [8]. Similarly, increased transcription of *cdkn1a* depends on Miz1 in response to DNA damage [9] as well as in models of cellular differentiation [13]–[15]. Miz1 also forms repressive complexes with the Bcl-6 and Gfi-1 oncoproteins. Both complexes are capable of repressing expression of *cdkn1a*, and, in the case of Gfi-1, also of *cdkn2b*. These observations suggest that Miz1 functions as a general mediator of repression in association with several transcription factors [13], [16], [17].

The constitutive knockout of Miz1 is lethal at embryonic day E7.5 [18]. We previously analysed the function of Miz1 in keratinocytes using a conditional Cre/lox knockout model, in which the Cre recombinase is targeted via the keratin 14 promoter to the basal layer of both intra- and interfollicular epidermis [19], where Miz1 is predominantly expressed [12]. In this model, loxP sites flank exons 3 and 4, which encode the POZ domain [2], and deletion of these exons results in expression of a truncated Miz1 protein lacking the POZ domain [20]. Consistent with the biochemical model described above, keratinocytes lacking the Miz1 POZ domain show an attenuated expression of Miz1 target genes in response to TGF-β [20]. Furthermore, animals homozygous for this deletion exhibit an impaired morphogenesis of hair follicles with irregular order and extended length of the follicle, formation of epidermal cysts, delayed catagen during the hair cycle, loss of zig-zag hairs, as well as the occurrence of pigment incontinence in older animals [20].

The model described above suggests that Miz1 has a repressive function in highly proliferative and tumor tissues that express high levels of Myc, which may not be revealed during normal development [21]. Recent work by Trumpp and colleagues demonstrated that endogenous Myc is required for the formation of skin papillomas. The critical function of endogenous Myc in this context is to repress expression of *cdkn1a*, since deletion of *c-myc* leads to a loss of tumor formation as well as elevated levels of p21^Cip1^, and co-deletion of *cdkn1a* fully restores tumor formation [22]. We now used the conditional Miz1-POZ domain knockout model to test the role of Miz1 in proliferation, differentiation and tumorigenesis in keratinocytes. We report here that the deletion of the Miz1 POZ domain leads to increased differentiation and reduced proliferation of keratinocytes when skin is challenged by the tumor promoter agent 12-O-tetradecanoylphorbol-13-acetate (TPA) as well as strongly decreased papilloma formation. These alterations are dependent on an altered regulation of *cdkn1a expression*. Our findings show that Miz1 is part of a repressor complex that is critical for restraining p21^Cip1^ expression in response to stimuli that enhance proliferation and promote skin carcinogenesis.

## Results

### The number of label retaining cells and the distribution of stem cell markers are unaffected in Miz1ΔPOZ mice

**Figure 1:**
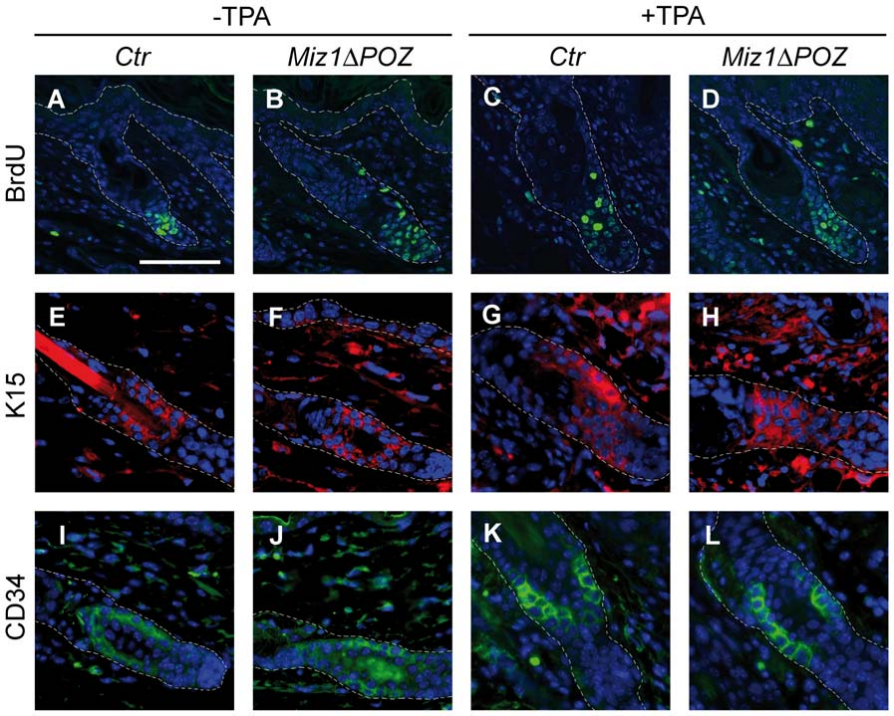
The skin stem cell compartment is unaltered in *Miz1*Δ*POZ* mice. Animals, which were labelled on day P10 with BrdU, showed label-retaining cells two weeks later, predominantly located in the bulge region of the hair follicle. No difference was observed between control (*Ctr*) and *Miz1*Δ*POZ* mice, neither without nor with TPA treatment (**A–D**). Skin stem cell markers Keratin 15 (K15; **E–H**) and CD34 (**I–L**) did not reveal differences between the different genotypes or treatments. Number of animals analysed: n = 3 for F–J and L; n = 4 for B, C and E; n = 5 for D and K; n = 6 for A. Bar: 50 µm.

The homozygous deletion of the Miz1 POZ domain in keratinocytes, using a mouse strain that expresses Cre recombinase under the control of the keratin 14 promoter (hereafter called *Miz1*Δ*POZ* mice; corresponding control animals do not express Cre recombinase; see also Material and Methods), revealed a complex skin phenotype [20]. To assess whether a defect of the stem cell compartment, located at the bulge region of the hair follicle, can account for the observed phenotypes in *Miz1*Δ*POZ* mice, we visualized label-retaining cells (LRCs; [23]) by injecting BrdU on day 10 post partum (P10) and analysed the number and location of LRCs on P24. No significant morphological difference in number and location of BrdU positive cells of the bulge region was detected comparing control and *Miz1*Δ*POZ* animals (Figure 1 A and B and Figure S1). To test whether enhanced proliferation has an impact on LRCs, we applied 12-O-tetradecanoylphorbol-13-acetate (TPA), a known enhancer of keratinocyte proliferation [24], once per day over five days. Again, no significant difference in number and location of LRCs of the bulge region was observed between control and *Miz1*Δ*POZ* animals (Figure 1 C and D and Figure S1).

In addition, immunohistochemical stainings for the stem cell markers K15 (Figure 1 E–H) and CD34 (Figure 1 I–L) [25], [26] revealed no difference in the number and location of labelled cells between control and *Miz1*Δ*POZ* animals, irrespective of TPA or control treatment. Our data indicate that the deletion of the *Miz1*Δ*POZ* domain has little effect on the location, number and proliferation of stem cells in the bulge region of *Miz1*Δ*POZ* mice.

### Alterations of differentiation and proliferation after TPA treatment are dependent on p21^cip1^

**Figure 2:**
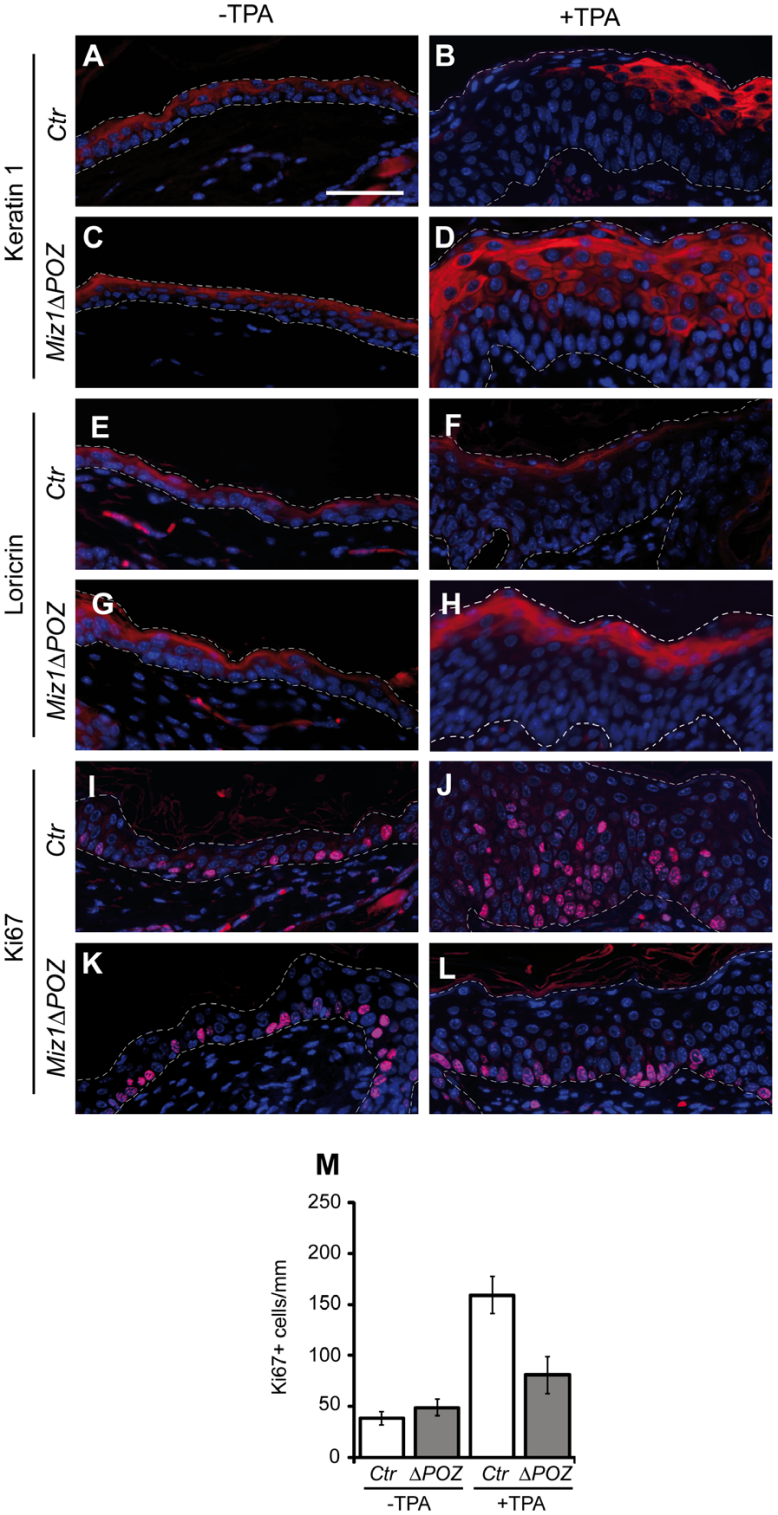
Epidermal differentiation and proliferation is altered upon TPA treatment. Immunohistochemical staining revealed no difference in the expression of the differentiation markers keratin 1 or loricrin (A, C and E, G) in the epidermis of untreated control (*Ctr*) or *Miz1*Δ*POZ* mice. When *Ctr* animals were treated with TPA, focal areas were observed lacking these differentiation markers (B, F). In contrast, such foci did not occur in the skin of *Miz1*Δ*POZ* mice (D, H). Immunohistochemistry for the proliferation marker Ki67 revealed positive cells in the basal cell layer of untreated skin in both genotypes (I, K) and the labelling index was not significantly different (M, −TPA; n = 5 for each genotype). After TPA treatment, the Ki67 labelling index in *Ctr* animals was about twice as high as in *Miz1*Δ*POZ* animals (M, +TPA; n = 5 for each genotype; *Ctr* vs *Miz1*Δ*POZ* for ± TPA: p<0.0001). In addition, Ki67 positive cells were scattered through the suprabasal cell layers of the epidermis in *Ctr* but not in *Miz1*Δ*POZ* animals (J, L). Bar: 50 µm.

Since Miz1, together with Myc, regulates the expression of genes encoding cyclin dependent kinase inhibitors like *cdkn2b* (encoding p15^Ink4b^) or *cdkn1a* (encoding p21^Cip1^) we next asked whether proliferation, differentiation and apoptosis of interfollicular keratinocytes are affected when a functional Miz1 protein is missing. The epidermis of control and *Miz1*Δ*POZ* mice showed no difference in the expression pattern of the differentiation markers keratin 1 (Figure 2 A and C), loricrin (Figure 2 E and G) or filaggrin (Figure S2F and H). Additionally, the number and location of cells positive for the proliferation marker Ki67 was unaltered (Figure 2 I, K and M). When mice were treated with TPA, the thickness of the epidermis increased as expected (Figure S2A–E), and the expression of the suprabasal differentiation markers keratin 1 and loricrin, but not filaggrin, was undetectable in large areas of the epidermis from control animals (Figure 2 B, F and Figure S2F and G). In contrast, thickening of the epidermis was slightly but significantly reduced in *Miz1*Δ*POZ* mice under TPA treatment (Figure S2E) and all three markers of differentiation remained prominent throughout the epidermis of *Miz1*Δ*POZ* mice (Fig. 2 D, H and Figure S2H and I). Furthermore, skin from *Miz1*Δ*POZ* mice exhibited keratin 1 staining in lower suprabasal cell layers, relative to control animals, where keratin 1 expression was mostly restricted to superficial epidermal cell layers (Figure 2 B and D). We conclude that treatment with TPA delays the differentiation of keratinocytes in control, but not in *Miz1*Δ*POZ* mice.

Consistent with these observations, application of TPA over five days significantly enhanced the number of Ki67 positive cells in the epidermis of control animals, but to a much lesser extent in the epidermis of *Miz1*Δ*POZ* animals (Figure 2 J, L and M). In addition, while a considerable number of Ki67 positive cells were located in suprabasal cell layers in control mice, this was not observed in *Miz1*Δ*POZ* mice (Figure 2 J, L), strongly indicating that the absence of the Miz1 POZ domain prevents cell cycle entry in response to TPA. Taken together, our findings show that a decrease of cell proliferation and an earlier onset of increased differentiation attenuate the effect of TPA in the epidermis of *Miz1*Δ*POZ* mice.

**Figure 3:**
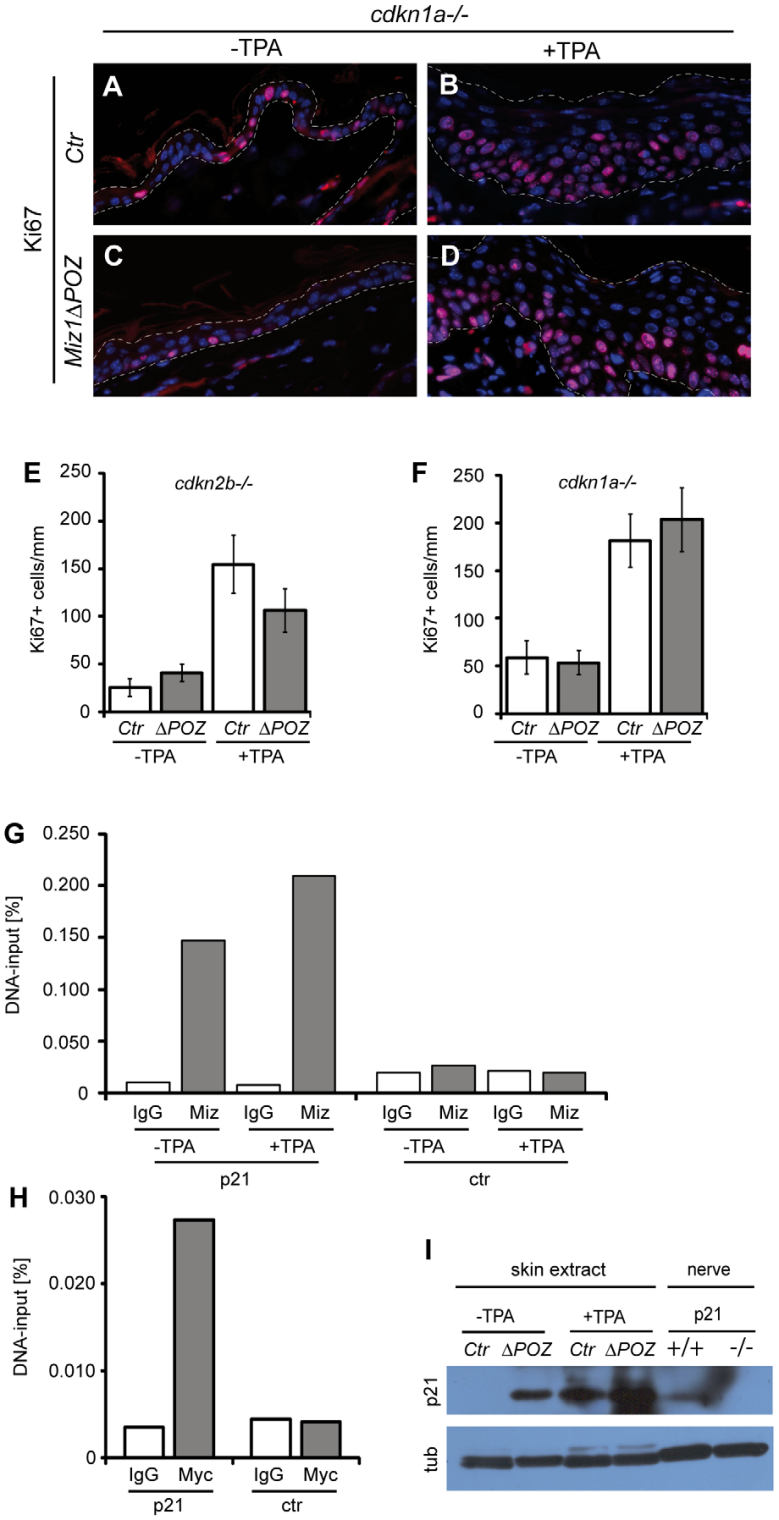
Altered proliferation in the skin of *Miz1ΔPOZ* mice depends on p21^Cip1^. TPA treatment of skin from control (*Ctr*) and *Miz1*Δ*POZ* mice on a *cdkn1a* null background shows the same increase of the Ki67 labelling index and scattering of Ki67 positive cells in the suprabasal layers of the epidermis (**A–D** and **F**; n = 3 for each genotype and condition; *Ctr* vs *Miz1*Δ*POZ* for −TPA: p = 0.4007; *Ctr* vs *Miz1*Δ*POZ* for +TPA: p = 0.4494). In contrast, the equivalent experiment on a *cdkn2b* null background (**E**; n = 3 for each genotype and condition) exhibited the same result as in *cdkn2b^+/+^* animals (compare with Figure 2 M; *Ctr* vs *Miz1*Δ*POZ* for ±TPA: p<0.0001). In *cdkn1a^+/+^* animals, p21^Cip1^ is upregulated in protein extracts from *Miz1*Δ*POZ*-mice (*ΔPOZ*) compared to extracts from control-mice (*Ctr*) either with or without TPA-treatment (**I**). Sciatic nerve extracts from *cdkn1a* ^+/+^ and *cdkn1a* ^−/−^ animals were used as positive and negative controls, respectively (see Material and Methods). Chromatin immunoprecipitation assay using chromatin from murine PAM212 keratinocytes and antibodies against Miz1 and Myc (**G**; **H**; one representative experiment of three independent experiments each). The experiment demonstrates that both transcription factors bind to the *cdkn1a* promoter in this cell type. Miz1 binds to the *cdkn1a* promoter in PAM212 keratinocytes, without and with TPA treatment (100 nM for 4 hours). Primers used either amplified genomic DNA comprising part of the *cdkn1a* promoter (p21) or a *cdkna1a* unrelated sequence of chromosome 17 (ctr). For details see Material and Methods. The graphs show the mean value of 2–3 technical replicas.

To genetically test whether one of the Miz1 regulated cyclin dependent kinase inhibitors, p15^Ink4b^ or p21^Cip1^, have a role in restricting proliferation and promoting differentiation of keratinocytes in *Miz1*Δ*POZ* mice, we generated *Miz1*Δ*POZ* mice that lack either *cdkn2b* or *cdkn1a*. TPA treatment of *Miz1ΔPOZ;cdkn2b^−/−^* mice revealed no difference to *Miz1ΔPOZ;cdkn2b^+/+^ mice* in regard to differentiation and proliferation of interfollicular keratinocytes, indicating that p15^Ink4b^ is not required for restraining proliferation of *Miz1*Δ*POZ* keratinocytes (Figure 3E, Figure S3A–D, E). In line with these findings we didn't observe changes in p15^ink4b^ expression by quantitative RT-PCR (data not shown). In contrast, keratinocyte proliferation was induced by TPA to the same extent in *Miz1*Δ*POZ*;*cdkn1a^−/−^* animals as in control animals (Figure 3A–D and F, Figure S3F). In addition, the extended focal ablation of differentiation markers that was observed in control animals also occurred in *Miz1*Δ*POZ*;*cdkn1a^−/−^* mice, in contrast to *Miz1*Δ*POZ* mice (Figure S4). These genetic data show that the impact of Miz1 on keratinocyte proliferation and differentiation depends on p21^Cip1^.

To determine the biochemical basis of these observations, we analysed p21^cip1^ expression by immunoblot analysis of skin from control and knockout animals with a *cdkn1a* ^+/+^ background (Fig. 3I). Without TPA treatment, expression of p21^cip1^ was below the limit of detection in the skin from control animals but gave a clear signal in skin from *Miz1*Δ*POZ* animals. TPA treatment induced the expression of p21^cip1^ in control animals and led to a further increase in p21^cip1^ expression in *Miz1*Δ*POZ* mice. Under both conditions p21^cip1^ expression was increased in *Miz1*Δ*POZ* animals compared to control animals, demonstrating directly that the Miz1 POZ domain restrains expression of p21^cip1^ *in vivo*.

To rule out the possibility that the increased p21^cip1^ expression was an indirect effect of an altered signal transduction in *Miz1*Δ*POZ* animals, we first analysed Myc levels and found by immunoblot analysis that addition of TPA elevated Myc levels to a similar extent in control and *Miz1*Δ*POZ* animals (Figure S5A). Second, we evaluated the activity of the Ras-Raf-MEK pathway via detection of phosphorylated ERK (p-ERK) [22]. Immunoblot analysis revealed a similar phosphorylation of ERK after TPA treatment in control and *Miz1*Δ*POZ* animals (Figure S5A). In addition, p-ERK was detected in the suprabasal cell layers of the epidermis independent of the genotype (Figure S5B–E). Third, analysis of expression of p53, a major regulator of *cdkn1a*, by immunoblot and immunohistochemistry revealed no evidence for a difference between *Miz1*Δ*POZ* and control animals (Figure S5A, F and G).

To confirm that *cdkn1a* is a direct target gene of Miz1 and Myc in keratinocytes, we performed chromatin immunoprecipitation (ChIP) assays with antibodies directed against Miz1 and Myc, respectively. Since the isolation of primary keratinocytes takes several hours under harsh conditions and since it is almost impossible to obtain sufficient primary keratinocytes for efficient chromatin isolation, we used the murine keratinocyte cell line PAM212 [27], which responds to TPA similarly as primary keratinocytes [28]. Using chromatin isolated from these cells, ChIP assays revealed that both Miz1 and Myc were bound to the core promoter region of *cdkn1a*, but not to a control region located about 20 Mb downstream of *cdkn1a*. The binding of Miz1 to the *cdkn1a* promoter was not altered under TPA treatment (Figure 3G and H).

Taken together our data show that (a) *cdkn1a* is a direct target gene of Myc and Miz1 in murine keratinocytes,(b) that the POZ domain of Miz1 is critical for repressing p21^Cip1^ expression *in vivo* and (c) that elevated levels of p21^Cip1^ restrain TPA-stimulated keratinocyte proliferation in *Miz1*Δ*POZ* mice.

### Reduced skin tumorigenesis in Miz1ΔPOZ mice

**Figure 4:**
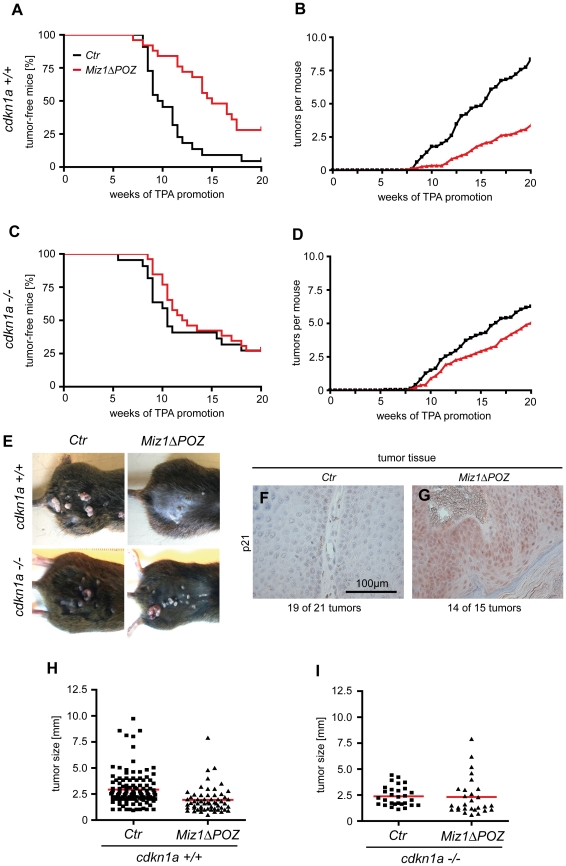
Formation of skin papillomas is decreased in *Miz1ΔPOZ* mice. (A) Animals with a conditional knockout of the *Miz1*Δ*POZ* domain in keratinocytes (n = 26) showed a reduced development of skin papillomas relative to control (*Ctr*) mice (n = 23; p<0.001). In addition, the average number of tumors per mouse was significantly reduced in *Miz1*Δ*POZ* animals compared to control animals (B). With a *cdkn1a* null background, tumor development (C) and average number of tumors per animal (D) in *Ctr* (n = 22) and *Miz1*Δ*POZ* (n = 26) mice were not significantly different (tumor development: p = 0.9789; tumors per animal: p = 0.1585). In (E), representative tumors of all genotypes are shown documenting a smaller size of tumors in *Miz1*Δ*POZ* mice with a *cdkn1a^+/+^* background. This is confirmed by the quantification of the tumor diameters (H; *Ctr* vs. *Miz1ΔPOZ:* p<0.0001). In contrast, tumor size on a *cdkn1a^−/−^* background was not significantly different between control and *Miz1*Δ*POZ* animals (I; *Ctr* vs. *Miz1*Δ*POZ*: p = 0.8788). When tumor tissue with a *cdkn1a^+/+^* background was stained with an antibody against p21^Cip1^, 14 out of 15 tumors from 9 *Miz1*Δ*POZ* mice exhibited a strong staining (G), while p21^Cip1^ expression was not detectable in 19 out of 21 tumors from 7 wild type mice (F) (see also Figure S8).

The reduced proliferation response to TPA treatment in the skin of *Miz1*Δ*POZ* animals led us to ask whether Miz1 plays a role in skin tumorigenesis. We applied the well-established two-stage skin carcinogenesis protocol using 7,2-dimethylbenz(a)anthracene (DMBA) as tumor initiator and 12-O-tetradecanoylphorbol-13-acetate (TPA) as tumor promoter [29]. Tumors initially emerged between weeks 8 and 9 of the TPA treatment both in control (see Materials and Methods) and in *Miz1*Δ*POZ* animals, indicating that the principal time course of tumor development is similar in both mouse strains (Figure 4A). However, whereas 50% of the control animals developed tumors between weeks 10 and 11 of TPA treatment (n = 23), it took 15–16 weeks until 50% of the *Miz1*Δ*POZ* animals exhibited tumors (Figure 4A; n = 26; p<0.001). To exclude that the tumors observed in *Miz1*Δ*POZ* mice developed from keratinocytes that have escaped Cre recombination, we isolated DNA from tumor samples and genotyped them by PCR. In all 45 tumors tested we could confirm efficient Cre-mediated recombination (Figure S6I). Since skin papillomas in this animal model are usually monoclonal [29], [30], a recombinant band indicates that the tumor has descended from a recombined keratinocyte. The non-recombined bands almost certainly come from cells of epidermal (melanocytes, dendritic cells) and/or dermal (fibroblasts, dendritic cells and many others) origin, in which the Cre recombinase is not active. This indicates that the tumors have not grown from escaper clones, but from cells lacking the Miz1 POZ domain. The gross morphology of tumors of comparable size from control and *Miz1*Δ*POZ* animals was identical. No difference in the pattern of outfoldings was observed. In both genotypes, the thicknesses of the epidermis and of the cornified layer, and the amount of keratohyalin granules were increased compared to the interfollicular epidermis. Finally, no spread of epidermal cells into the dermal compartment occurred (Figure S6A–H).

The decreased tumorigenesis in *Miz1*Δ*POZ* mice was further reflected by a reduced tumor burden per mouse, since the number of tumors was significantly lower in *Miz1*Δ*POZ* compared to control animals (*Miz1*Δ*POZ*: n = 3.38±4.30 tumors per mouse measured in 26 mice; control: n = 8.35±5.16 tumors per mouse measured in 23 mice; p<0.001; Figure 4B). Furthermore, tumors at the end of TPA treatment were significantly smaller in *Miz1*Δ*POZ* mice than in control mice (1.94±1.64 mm vs 2.93±1.73 mm; Figure 4E and H). To exclude that the reduced tumor size is caused by increased apoptosis, we performed a TUNEL assay. TUNEL positive cells were rarely found in the tumors of both genotypes and were almost absent in the interfollicular epidermis (Figure S7A), indicating that the tumor size in *Miz1*Δ*POZ* mice is not affected by increased programmed cell death. Finally, when TPA treatment was finished after 20 weeks and mice were subsequently observed for further 17 weeks, tumor diameter increased about threefold in control animals but remained constant in *Miz1*Δ*POZ* mice (Figure S7B and C). We conclude that tumor development and growth is strongly reduced in *Miz1*Δ*POZ* mice.

Strikingly, immunohistochemistry of papillomas revealed low levels of p21^cip1^ in keratinocytes from 19 out of 21 tumors from control animals, but high p21^cip1^ levels in keratinocytes from 14 out of 15 tumors of *Miz1*Δ*POZ* mice (Figure 4F and G; Figure S8A). Additionally, increase of p21^cip1^ in papillomas from *Miz1*Δ*POZ* mice was observed by immunoblot analysis (Figure S8B). To test the impact of p21^cip1^ genetically, we monitored tumor development in a cohort of *Miz1ΔPOZ;cdkn1a* ^−/−^ mice. In these experiments, we noted that the tumor burden per mouse in *cdkn1a^−/−^* control animals (Figure 4D) was lower than in *cdkn1a^+/+^* control animals (Figure 4B), most likely due to subtle differences in the overall genetic background of the animals used in the two experiments (see Materials and Methods) or possibly to a general lower tumor incidence in p21^cip1^ deficient animals [31],[32]. Importantly, *Miz1ΔPOZ;cdkn1a* ^−/−^ animals developed tumors with a time course that was indistinguishable from control *cdkn1a* ^−/−^ animals (Figure 4C; p = 0.6933). Furthermore, the difference of tumor burden between *cdkn1a^−/−^;Miz1ΔPOZ* and their corresponding control mice was smaller (5.04±7.03 vs 6.27±5.74 tumors per mouse, measured in 22 control and 26 *cdkn1a^−/−^;Miz1ΔPOZ* mice; p = 0.5139) than in an *cdkn1a^+/+^* background (8.35 vs 3.38 tumors per mouse, see above). Finally, there was no difference in the tumor size between control and *Miz1*Δ*POZ* animals in a *cdkn1a^−/−^* background (Figure 4I), in contrast to control and *Miz1*Δ*POZ* animals with a *cdkn1a^+/+^* background (Figure 4H). Taken together, we conclude that the reduced tumorigenicity observed in *Miz1*Δ*POZ* mice depends on the upregulation of p21^Cip1^ expression.

## Discussion

One well characterized function of Miz1 is the regulation of the cyclin dependent kinase inhibitor genes *cdkn2b* (encoding p15^Ink4b^), *cdkn1a* (encoding p21^Cip1^) and *cdkn1c* (encoding p57^Kip2^) [33], although a number of other genes are now known to be expressed in a Miz1-dependent manner [6], [11], [12]. The current model proposes that Miz1, complexed with nucleophosmin, binds to the core promoter of its target genes to enhance gene expression. Transactivation is blocked when the Myc/Max complex binds to Miz1, thereby releasing nucleophosmin [34], [35]. In this model, Miz1 has a dual role in expression of its target genes. In the absence of Myc, Miz1 contributes to target gene activation. However, Miz1 also serves to recruit Myc leading to the formation of a repressive complex. This suggests that abrogation of Miz1 function will enhance target gene expression in conditions of high Myc expression. The allele of Miz1 used here lacks the POZ domain, which is necessary both for the transactivating properties of Miz1 [1], [9] and for its stable association with chromatin [6], [21].

We observed fewer and smaller tumors in *Miz1*Δ*POZ* animals. A detailed analysis revealed that a reduced proliferation of keratinocytes in response to the tumor promoter, TPA, can account for the reduction of tumor growth in these animals, while changes in the stem cell compartment could not be uncovered. Interestingly, proliferation was completely restored in a *cdkn1a* null background, strongly suggesting that the increase in p21^Cip1^ expression that is observed in *Miz1*Δ*POZ* mice is responsible for the observed phenotype. This notion is further supported 1) by an immunoblot analysis revealing elevated p21^cip1^ levels in the skin from *Miz1*Δ*POZ* mice compared to their wildtype counterparts (Fig. 3I), 2) by immunohistochemical data showing that p21^Cip1^ is consistently expressed in tumors of *Miz1*Δ*POZ* mice, but is hardly detectable in control tumors (Fig. 4F, G) and 3) by the genetic experiment exhibiting a rescue of the reduced proliferation reflected by Ki67 positive cells (Figure 3A–D and F), a rescue of the tumor development (Figure 4C) and partial rescue of the tumor burden (Figure 4D) in *Miz1*Δ*POZ* mice on a *cdkn1a* null background. In contrast, *Miz1*Δ*POZ* mice with a *cdkn2b* null background exhibited only a small and statistically insignificant increase in Ki67 positive cells (Figure 3E; Figure S3A–E), suggesting that upregulation of p15^Ink4b^ is not involved in restraining proliferation of *Miz1*Δ*POZ* keratinocytes in response to TPA treatment.

While our data show that Miz1 has a critical role in repressing *cdkn1a* expression during skin carcinogenesis, they do not directly address the mechanism by which Miz1 acts in this system. For example, Miz1 has been suggested to associate with p53 [36]. While deletion of the POZ domain does not affect levels of p53 (Figure S5A), it is possible that it enhances p53 function in more subtle ways, leading to enhanced p21^cip1^ expression. Furthermore, we do not know which of the several oncoproteins that can repress transcription via Miz1 (see Introduction) are functional during skin carcinogenesis. However, our data can account for the results obtained using tamoxifen inducible *c-myc* ^−/−^ mice [22]. In these mice, DMBA/TPA treatment led to elevated expression of p21^Cip1^ and skin tumors could only be induced in the absence of p21^Cip1^, demonstrating that endogenous Myc has a critical function in repressing *cdkn1a* during skin tumor development. It should be noted, that a related model in which *c-myc* is deleted during development by a constitutively active Cre recombinase expressed under the keratin 5 promoter shows more severe phenotypes, suggesting that Myc has functions in addition to repressing p21^cip1^ during skin development [37].

Our data extend observations on Myc-induced lymphomagenesis in mice expressing a mutant allele of Myc that is selectively deficient in binding to Miz1 (MycV349D). Mice expressing this mutant display a reduced tumorigenesis, at least in part because binding of Myc to Miz1 is required to restrain expression of p15^Ink4b^ and of p57^kip2^ in the lymphomas [38]. Importantly, lymphomas arising in these mice showed an accumulation of senescent cells, suggesting that binding of Myc to Miz1 may be required to antagonize senescence during tumorigenesis.

p21^Cip1^ is a key player during the induction of senescence of human fibroblasts [39], keratinocytes [40], melanocytes [41] and mammary epithelial cells [42]. While we did not detect senescent cells in tumors that arose in either wild type or *Miz1*Δ*POZ* animals, a fraction of interfollicular and follicular keratinocytes and approximately 25% of the hair follicles stain positive for the senescence marker SA-ß-galactosidase in aged skin of *Miz1*Δ*POZ* but not of control animals (Figure S9; [43]). We suggest, therefore, that repression via Miz1 may be more broadly involved in suppressing senescence and the reduced tumorigenesis in *Miz1*Δ*POZ* mice may reflect the need to overcome p21^Cip1^ mediated senescence during tumor formation [44], [45]. While this remains to be formally demonstrated, the current data strongly support the view that the formation of a functional Miz1/Myc complex results in a context-dependent and cell type-specific attenuation, or even abrogation, of critical growth arrest pathways during tumorigenesis.

## Materials and Methods

### Transgenic mice

*Miz1* ^lox/lox^ mice [20] were crossed with K14Cre mice [19] to generate a conditional knockout of the POZ domain of Miz1 in murine basal epidermal cells as described elsewhere [20]. Mice were backcrossed 6 times on a 129S2/SvHsd background. Here, mice which are K14*cre* ^+^;*Miz1^flox/flox^* are designated *MizΔPOZ*-mice, while K14*Cre* ^−^;*Miz1^flox/flox^* mice were used as control animals designated *Ctr*. *MizΔPOZ* mice were crossed on a *cdkn2b* [46] and *cdkn1a* [47] deficient background, here designated *MizΔPOZ;cdkn2b^−/−^* and *MizΔPOZ;cdkn1a^−/−^* mice, respectively. *cdkn2b^−/−^* animals, with a FVB background, were a generous gift from A. Burns, Amsterdam. *cdkn1a^−/−^* animals, with a 129S4/SvJae genetic background, were purchased from Jackson laboratory (stock no. 008184).

49 d old mice were treated on 5 consecutive days with 5 nmol TPA (Sigma) in 100 µl acetone. TPA was locally applied on the dorsal skin, which was shaved 1 week before the first treatment. The mice were sacrificed 24 h after the last treatment.

In the 2-stage tumorigenesis experiment [29], 100 nmol DMBA (Sigma) in 100 µl acetone was once applied on the shaved dorsal skin of 49 d old mice. 1 week after the DMBA-application, the TPA treatment was started. Mice were then treated with 5 nmol TPA in 100 µl acetone for 20 weeks with two TPA applications per week.

Label-retaining cells (LCRs) were demonstrated in a bromodesoxyuridine (BrdU) pulse-chase experiment. On day 10 post partum (P10), 10 mg BrdU dissolved in 50 µl sterile PBS were injected intraperitoneally 5 times once every hour. On days P18 to P23 animals were treated with TPA as described above and the skin was prepared for histology. Immunocytochemical staining of BrdU was performed as described below.

Research involving mice have been conducted according to the German Animal Protection Law (Tierschutzgesetz). The application for the experiments was reviewed and approved by the responsible local authorities (Regierungspraesidium Giessen, reference numbers V 54 - 19 c 20/15 (1) MR20/10 Nr. 23/2005, V 54 - 19 c 20/15 (1) MR20/10 Nr. 95/2009 and V 54 - 19 c 20/15 (1) MR20/10 Nr. 66/2010)

### Immunoblot analysis

Protein samples were extracted from dorsal skin and homogenized in RIPA buffer, containing 1% Triton X-100; 1% sodium deoxycholate; 0,1% SDS; 150 mM NaCl; 10 mM Na_2_HPO_4_; 2 mM EDTA; 1% Apronitin; 50 mM NaF; 200 mM Na_3_VO_4_; pH 7,4. As a positive control for p21^cip1^, extracts from sciatic nerves were used [48]. Protein concentration was determined using the BCA-assay (Sigma). 20 µg per sample were separated on 10% polyacrylamide-SDS gels according to standard procedures. Proteins were blotted on nitrocellulose membranes and the blots were stained with antibodies against the following proteins: c-Myc (N-262, Santa Cruz; 1∶400), p21 (C-19, Santa Cruz; 1∶100), p-ERK (T202/Y204, Cell Signalling; 1∶200), p53 (FL-393, Santa Cruz; 1∶400), tubulin (YL1/2, Abcam; 1∶2000) at 4°C overnight or for 72 hours in case of p53. Appropriate secondary peroxidase labelled antibodies (Biorad) were applied 1 hour at room temperature and antibody binding was visualized using the Lumi-Light Western blotting substrate from Roche.

### Chromatin immunoprecipitation assay

Chromatin immunoprecipitation was carried out according to Boyd [49] using the mouse keratinocyte cell line PAM212 [27]. Briefly, the chromatin was sonicated at 4°C using a Bioruptor™ NextGen (Diagenode) for 15×30 sec at 20 Hz and 320 W with 30 sec breaks between each sonication step. The Immunoprecipitation Starter Pack (GE Healthcare) was used with antibodies directed against c-Myc (N-262; Santa Cruz) and Miz1 (10E2, Staller *et al.* 2001). For the isotype control, IgG from mouse and rabbit serum (Sigma) was used. After the crosslink reversion, the chromatin was purified with the QIAquick PCR Purification Kit (QIAGEN).

The promoter binding was detected by qPCR on a Mx3005p PCR machine (Stratagene/Agilent) with a QPCR SYBR Green Mix (Thermo Scientific), using CTCAGCTCTAACTGTACTGTTGTTCA as forward and CTGGGCTATTCTCTTGTCACG as reverse primer, to detect the *cdkn1a* promoter sequence by amplifying genomic DNA between basepairs 29.230.454–29.230.529 of chromosome 17. Control primers were TCATCCCACCCAGGAGTATT as forward primer and GAGTACATTTAACCAACTATCAGAGCA as reverse primer, respectively, amplifying genomic DNA between basepairs 9.600.536–9.600.628 of chromosome 17 being unrelated to *cdkn1a*.

### Histology

Skin samples were fixed in PBS buffered 3.7% formaldehyde and embedded in paraffin according to standard procedures. For immunohistochemistry, 3 µm sections were applied on silane-coated slides, preincubated with 10% goat serum (Sigma) and, if necessary, further treated as outlined below. For Ki67, p21^Cip1^ and p-ERK staining, slides were microwaved in 10 mM citrate buffer pH 6 for 3×5 min. For bromodesoxyuridine (BrdU) staining, slides were incubated for 30 min in 2 N HCl/0.5% Triton X-100, for 3 min in borax buffer (0.5 M sodium diborate/0,5 M boric acid, pH = 7.6) at RT and for 3 min in 0.025% trypsin in 0,05 M Tris/HCL, pH = 7.4. Primary antibodies were diluted in 10% goat serum (Dako) and incubated at 4°C overnight. Antibodies against the following antigens were used: Ki67 (Dako; 1∶50), BrdU (Dianova; 1∶100), p-ERK (T202/Y204, Cell Signalling; 1∶100), CD34 (BD; 1∶100), K15 (Abcam; 1∶100), p21^Cip1^ (Abcam; 1∶100), keratin 1 (Covance, 1∶1000), loricrin (Covance; 1∶1000). For visualization, appropriate secondary antibodies labelled either with FITC, TRITC (Molecular Probes) or with peroxidase were used. Slides were incubated 1 hour at room temperature and were subsequently covered with Mowiol. For documentation, a motorized BX61 microscope (Olympus) equipped with a F-View digital camera was used (Soft Imaging System, Münster, Germany).

The TUNEL assay was performed using the DeadEnd kit (Promega) according to manufacturer instructions. The staining for SA-ß-galactosidase was performed as described by Dimri et al. [43]. To ascertain the percentage of positive hair follicles, about 100 follicles per sample were counted.

### Morphometric analysis

The number of Ki67 positive cells per mm of skin and the ratio of suprabasal Ki67 positive cells were measured using the program cell^F^ (Olympus). From 3 to 5 mice per condition (control, *MizΔPOZ*, *MizΔPOZ;cdkn2b^−/−^* and *MizΔPOZ;cdkn1a^−/−^*; TPA-treated and untreated) 15–25 pictures were taken. In each picture, the length of the epidermis was measured and the related Ki67 positive basal and suprabasal cells were counted. The amount of all Ki67 positive cells per mm of skin was calculated. In addition, the ratio of basal to superbasal Ki67 positive cells was determined.

### Statistical analysis

Mean values and standard deviations of the morphometric and ChIP data were calculated with Excel (Microsoft). The statistical significance of the morphometric data, the Kaplan-Meier estimator and the average number of tumors per mouse was calculated using the Student's *t*-test as implemented in the program GraphPad Prism (GraphPad Software).

## Supporting Information

Figure S1 **Label-retaining cells (LRCs) in the bulge region.** (A) Documentation of LRC number variability in bulge regions from ctr and *Miz1*Δ*POZ* animals, without and with TPA treatment. In (B), the percentage of LRCs (% BrdU positive cells) counted in the bulge region area are shown. 19 to 25 bulge regions per condition were evaluated for BrdU positive cells.
(TIF)

Figure S2 **TPA treated control and** ***Miz1ΔPOZ*** **epidermis.** HE-staining of control (A, B) and *Miz1*Δ*POZ* epidermis (C, D) under TPA treatment (B, D) or in untreated skin (A, C). The size of scale bar in A is 50 µm. The average epidermal thickness of TPA treated and untreated control and *Miz1*Δ*POZ* epidermis is shown in E. 100 single measurements per animal were done with 3 animals per condition. Fluorescence staining of filaggrin in control (F, G) and *Miz1*Δ*POZ* (H, I) skin with and without TPA treatment (+/−TPA). Filaggrin is equally expressed in *Ctr* and *Miz1*Δ*POZ* suprabasal epidermis, either with or without TPA treatment. Percentage of suprabasal Ki67 positive keratinocytes in untreated and TPA treated *Ctr* and *Miz1*Δ*POZ* skin (J).
(TIF)

Figure S3 **TPA treated control and** ***Miz1ΔPOZ*** **epidermis with a** ***cdkn2b^−/−^*** **background.** Fluorescence staining of Ki67 in *Ctr* (A, B) and *Miz1*Δ*POZ* (C, D) skin with a *cdkn2b* (encoding p15^INK4b^) deficient background with and without TPA treatment (+/−TPA). The additional deletion of *cdkn2b* does not rescue the reduced proliferation in TPA treated *Miz1*Δ*POZ* skin compared to TPA treated *Ctr* skin. Quantification of suprabasal Ki67 positive keratinocytes in untreated and TPA treated *Ctr* and *Miz1*Δ*POZ* skin with either a p15^INK4b^ (E) or a p21^cip1^ (F) deficient background. Under TPA treatment, suprabasal Ki67 positive cells are significantly reduced in *Miz1*Δ*POZ* skin compared to *Ctr* skin in mice with a *cdkn2b^−/−^* background (E; p<0.0001), as observed in *cdkna2b^+/+^* animals (compare with Figure S2 J). In contrast, a complete rescue was achieved in *cdkn1a^−/−^* animals where no difference of Ki67 suprabasal cells was observed between control and *Miz1*Δ*POZ* mice (F; p = 0.9316).
(TIF)

Figure S4 **Differentiation in** ***cdkn2b*** **or** ***cdkn1a*** **deficient** ***Miz1ΔPOZ*** **epidermis.** Fluorescence staining of keratin 1 (A–H) and loricrin (I–P) in control and *Miz1*Δ*POZ* skin with and without TPA treatment (+/−TPA) either with a *cdkn2b* (A–D and I–L) or *cdkn1a* (E–H and M–P) deficient background. In *cdkn2b^−/−^* mice and upon TPA treatment, keratin 1 expression is focally interrupted in *Ctr* skin while *Miz1*Δ*POZ* skin shows continuous keratin 1 expression (B, D). With a *cdkn1a* deficient background, *Ctr* and *Miz1*Δ*POZ* skin both show a focal interruption of keratin 1 expression after TPA (F, H). Also, with a *cdkn2b^−/−^* background, loricrin expression is focally reduced in *Ctr* skin (J) but not in *Miz1*Δ*POZ* skin (L), while in *cdkn1a^−/−^* skin, focal reduction of loricrin expression can be observed in both *Ctr* (N) and *Miz1*Δ*POZ* skin (P). The described expression patterns of keratin 1 and loricrin only occurred in TPA treated skin, whereas untreated skin did not show differences between *Ctr* and *Miz1*Δ*POZ* animals in regard to keratin 1 and loricrin expression, neither with a *cdkn2b* (A, C, I, K), nor with a *cdkn1a* deficient background (E, G, M, O).
(TIF)

Figure S5 **ERK-phosphorylation, c-Myc and p53 expression in** ***Miz1ΔPOZ*** **epidermis.** Immunoblot of phosphorylated ERK (p-ERK), c-Myc and p53 in extracts of murine Control (*Ctr*) and *Miz1*Δ*POZ* skin (A), untreated or treated with TPA (−/+TPA). α-tubulin was used as a loading control. The expression of p-ERK was also visualized in murine epidermis via immunohistochemistry in control (B, C) and *Miz1*Δ*POZ* samples (D, E) both untreated (B, D) or TPA treated (C, E). Furthermore, p53 stained by immunohistochemistry in tumors did not reveal a difference between control (*Ctr*) and *Miz1*Δ*POZ* papillomas (F, G), in contrast to p21^cip1^ expression (see Figure 4F, G and Figure S8).
(TIF)

Figure S6 **Histology and genotyping of** ***Miz1ΔPOZ*** **papillomas.** HE-staining of control (A, C, D, G) and *Miz1*Δ*POZ* papillomas (B, E, F, H). The length of the scale bars is 600 µm in A and B, 100 µm in C–F and 50 µm in G and H. (I) Miz1 genotyping of murine tail skin (S) and tumor tissue (T). A control (*Ctr*) animal and one tumor with a floxed *Miz1* allele and no Cre recombinase expression was genotyped as a negative control. Animals 1–11 are *Miz1*Δ*POZ* animals with a floxed *Miz1* allele which express Cre recombinase. The lower band at 180 bp indicates the recombinant allele, while the upper band at 311 bp indicates the floxed allele. A floxed allele can also be detected in tumors from *Miz1*Δ*POZ* animals due to the presence in the samples of other (non-keratinocyte) epidermal and dermal cell types that do not express Cre recombinase.
(TIF)

Figure S7 **Apoptosis and tumor growth in tissue from control (ctr) and** ***Miz1ΔPOZ*** **animals.** (A) While there were essentially no TUNEL positive cells in the interfollicular skin, we occasionally observed TUNEL positive cells in tumors independent of the genotype, although most tumors from both genotypes lacked TUNEL positive cells. As a positive control for the assay we used either skin fixed in Carnoy's solution, where most nuclei should be positive because of an acidic hydrolysis of the DNA (due to the acetic acid which is a component of this fixative) or thymus which usually exhibits a large number of apoptotic T-cells, predominately in the cortex. (B) Tumor development during 17 weeks after the last TPA treatment. Representative pictures of control (*Ctr*) and *Miz1*Δ*POZ* papillomas 17 weeks after the last TPA treatment (B). Measurement of the tumor diameter (C) revealed an increased tumor-size in *Ctr* animals but not in *Miz1ΔPOZ mice* (compare with Figure 4H).
(TIF)

Figure S8 **p21-expression in** ***Miz1ΔPOZ*** **papillomas.** Immunohistochemistry of control (*Ctr*) and *Miz1*Δ*POZ* papillomas showing the expression of p21^cip1^ (A). Each slide indicates a representative region of an individual tumor. In the majority of *Miz1*Δ*POZ* papillomas, p21^cip1^ was upregulated, whereas in most *Ctr* tumors p21^cip1^ expression was not detectable. Expression of p21 protein in papillomas was also analyzed by immunoblot (B) in each of three (1–3) *Ctr* and *Miz1*Δ*POZ* papillomas with p21^+/+^. *Ctr* and *Miz1*Δ*POZ* tumor samples with a p21^−/−^ background are negative controls. All *Miz1*Δ*POZ*-tumors have an increased p21 expression compared to *Ctr* tumors, while in p21^−/−^ tumors, no p21 expression was detectable.
(TIF)

Figure S9 **Tumors of** ***Miz1ΔPOZ*** **mice are not positive for SA-ß-galactosidase.** Tumors from control (*Ctr*) (A) and *Miz1*Δ*POZ* (B) animals after 20 weeks of TPA treatment were histochemically stained for SA-ß-galactosidase, but were not positive independent of the genotype. In contrast, skin from one year old *Miz1ΔPOZ mice* displayed a focal staining which was absent in *Ctr* animals (C, D). In addition, about 25% of hair follicles stained positive for SA-ß-galactosidase in *Miz1*Δ*POZ* but not in *Ctr* animals (E). Arrowheads indicate sebaceous glands, which stain always positive for SA-ß-galactosidase.
(TIF)
