# Supplementary figures and images for: Miz1 Is a Critical Repressor of cdkn1a during Skin Tumorigenesis
Source: PLoS One. 2012 Apr 11;7(4):e34885. doi: 10.1371/journal.pone.0034885 (PMC3324535; doi:10.1371/journal.pone.0034885)

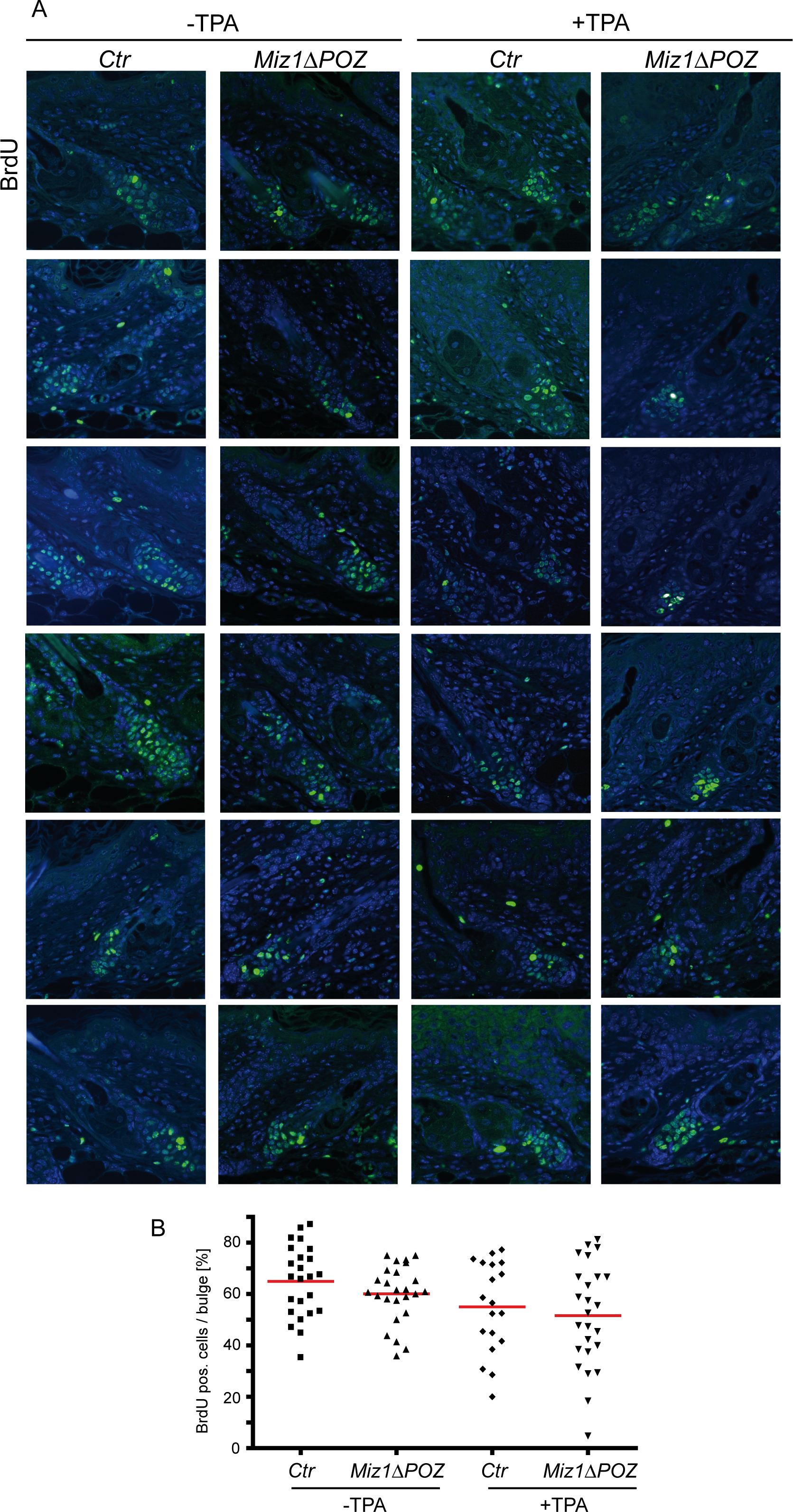

Supplement: Figure S1 — Label-retaining cells (LRCs) in the bulge region. (A) Documentation of LRC number variability in bulge regions from ctr and Miz1ΔPOZ animals, without and with TPA treatment. In (B), the percentage of LRCs (% BrdU positive cells) counted in the bulge region area are shown. 19 to 25 bulge regions per condition were evaluated for BrdU positive cells. (TIF) [file pone.0034885.s001.tif]

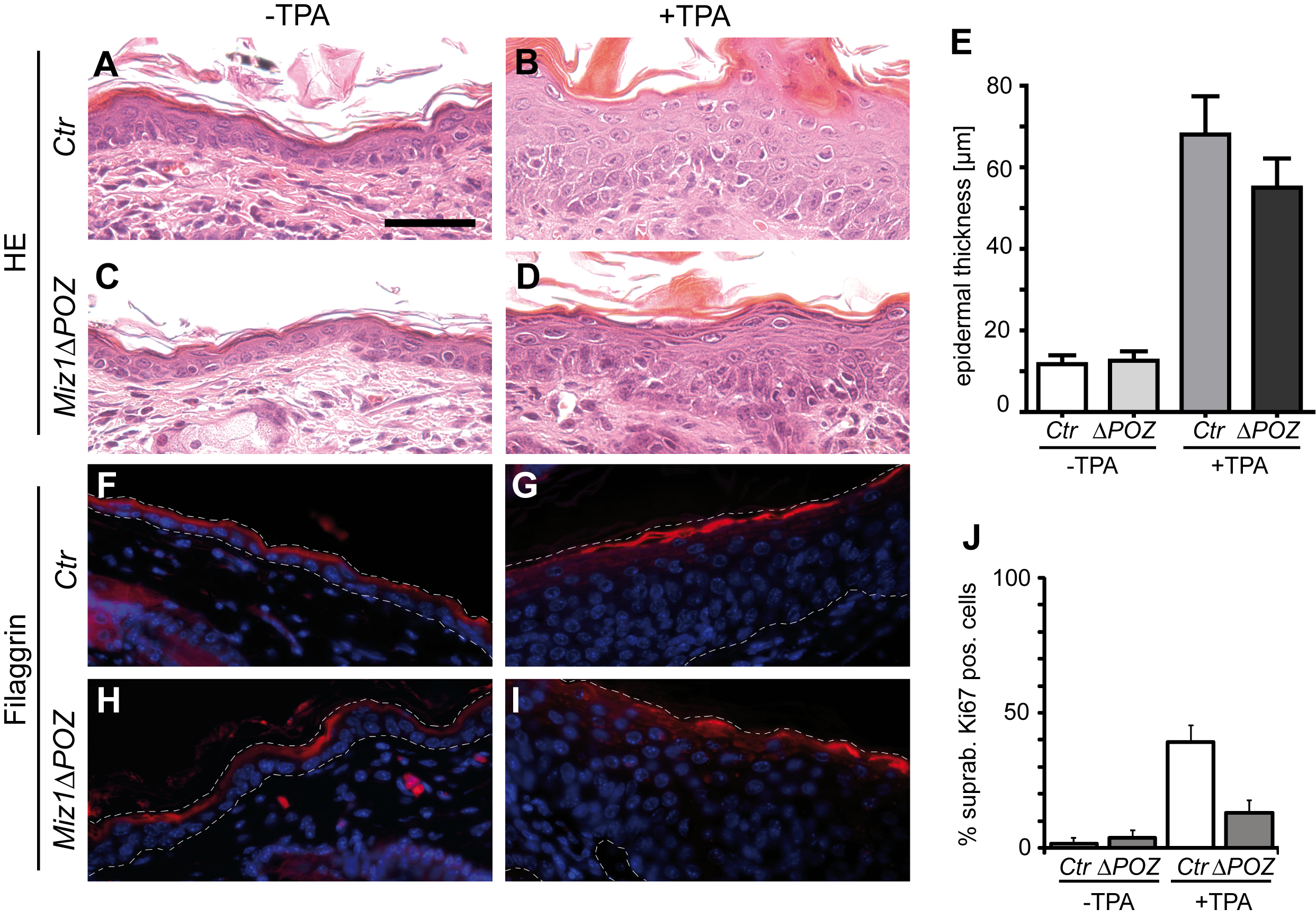

Supplement: Figure S2 — TPA treated control and Miz1ΔPOZ epidermis. HE-staining of control (A, B) and Miz1ΔPOZ epidermis (C, D) under TPA treatment (B, D) or in untreated skin (A, C). The size of scale bar in A is 50 µm. The average epidermal thickness of TPA treated and untreated control and Miz1ΔPOZ epidermis is shown in E. 100 single measurements per animal were done with 3 animals per condition. Fluorescence staining of filaggrin in control (F, G) and Miz1ΔPOZ (H, I) skin with and without TPA treatment (+/−TPA). Filaggrin is equally expressed in Ctr and Miz1ΔPOZ suprabasal epidermis, either with or without TPA treatment. Percentage of suprabasal Ki67 positive keratinocytes in untreated and TPA treated Ctr and Miz1ΔPOZ skin (J). (TIF) [file pone.0034885.s002.tif]

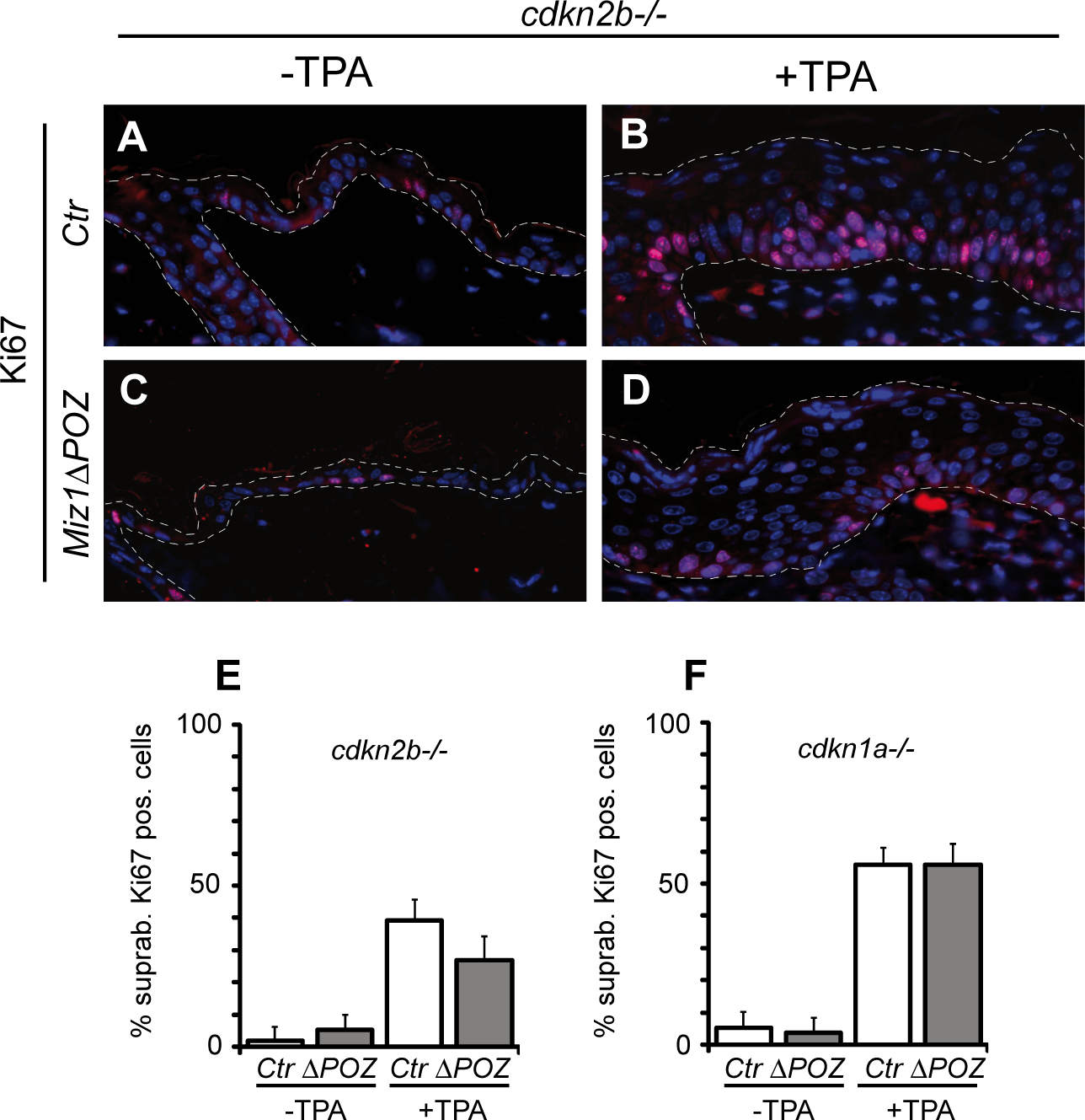

Supplement: Figure S3 — TPA treated control and Miz1ΔPOZ epidermis with a cdkn2b−/− background. Fluorescence staining of Ki67 in Ctr (A, B) and Miz1ΔPOZ (C, D) skin with a cdkn2b (encoding p15INK4b) deficient background with and without TPA treatment (+/−TPA). The additional deletion of cdkn2b does not rescue the reduced proliferation in TPA treated Miz1ΔPOZ skin compared to TPA treated Ctr skin. Quantification of suprabasal Ki67 positive keratinocytes in untreated and TPA treated Ctr and Miz1ΔPOZ skin with either a p15INK4b (E) or a p21cip1 (F) deficient background. Under TPA treatment, suprabasal Ki67 positive cells are significantly reduced in Miz1ΔPOZ skin compared to Ctr skin in mice with a cdkn2b−/− background (E; p<0.0001), as observed in cdkna2b+/+ animals (compare with Figure S2 J). In contrast, a complete rescue was achieved in cdkn1a−/− animals where no difference of Ki67 suprabasal cells was observed between control and Miz1ΔPOZ mice (F; p = 0.9316). (TIF) [file pone.0034885.s003.tif]

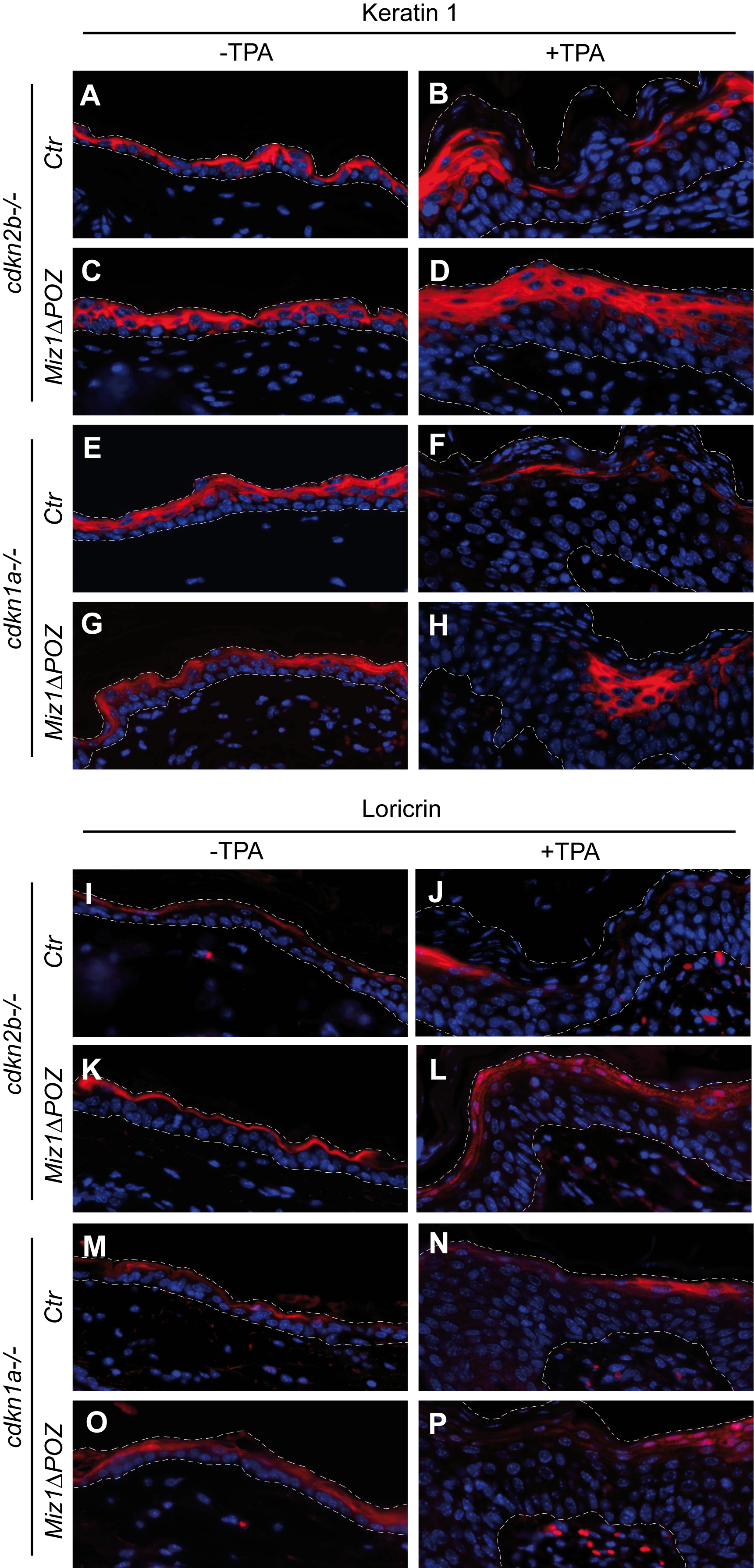

Supplement: Figure S4 — Differentiation in cdkn2b or cdkn1a deficient Miz1ΔPOZ epidermis. Fluorescence staining of keratin 1 (A–H) and loricrin (I–P) in control and Miz1ΔPOZ skin with and without TPA treatment (+/−TPA) either with a cdkn2b (A–D and I–L) or cdkn1a (E–H and M–P) deficient background. In cdkn2b−/− mice and upon TPA treatment, keratin 1 expression is focally interrupted in Ctr skin while Miz1ΔPOZ skin shows continuous keratin 1 expression (B, D). With a cdkn1a deficient background, Ctr and Miz1ΔPOZ skin both show a focal interruption of keratin 1 expression after TPA (F, H). Also, with a cdkn2b−/− background, loricrin expression is focally reduced in Ctr skin (J) but not in Miz1ΔPOZ skin (L), while in cdkn1a−/− skin, focal reduction of loricrin expression can be observed in both Ctr (N) and Miz1ΔPOZ skin (P). The described expression patterns of keratin 1 and loricrin only occurred in TPA treated skin, whereas untreated skin did not show differences between Ctr and Miz1ΔPOZ animals in regard to keratin 1 and loricrin expression, neither with a cdkn2b (A, C, I, K), nor with a cdkn1a deficient background (E, G, M, O). (TIF) [file pone.0034885.s004.tif]

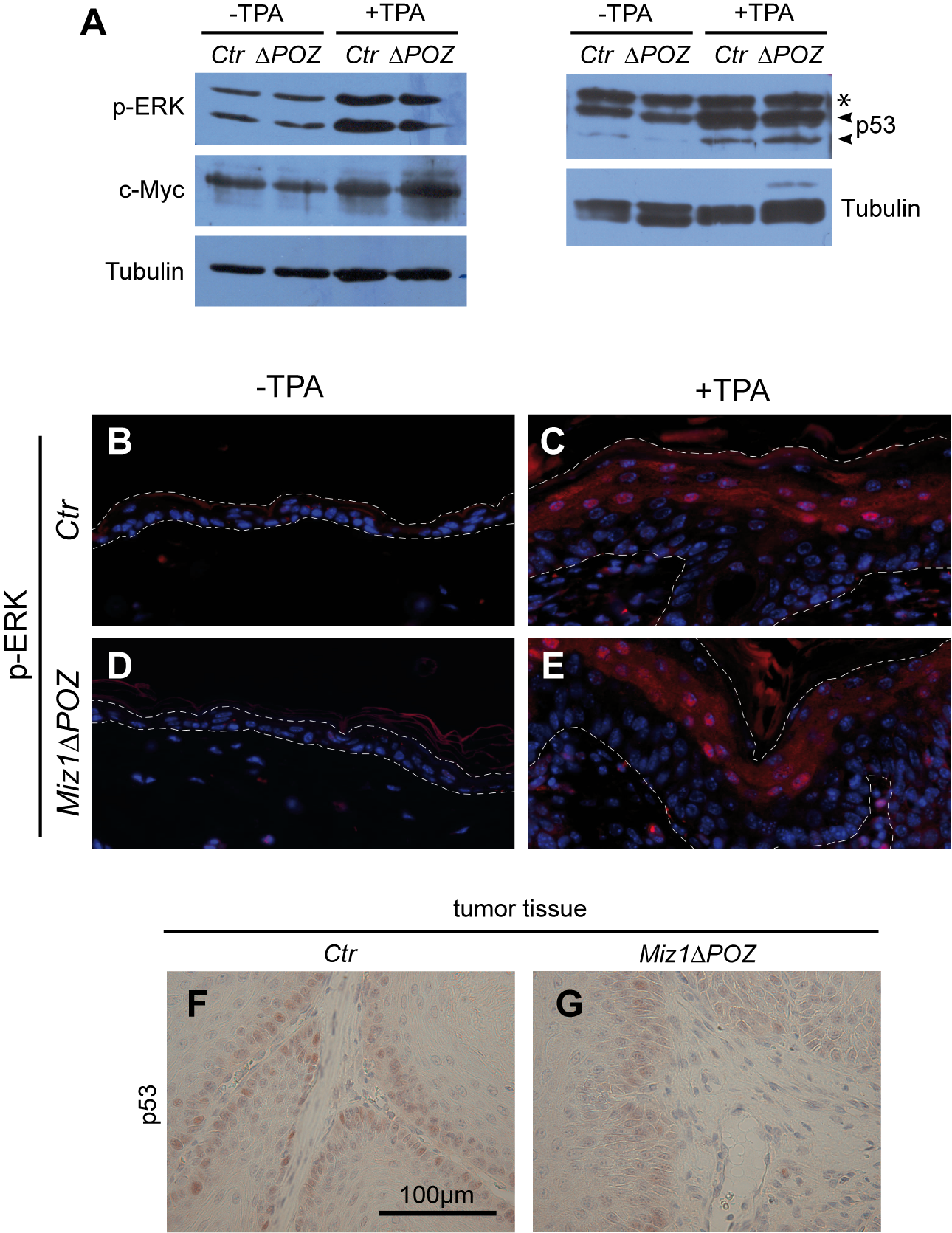

Supplement: Figure S5 — ERK-phosphorylation, c-Myc and p53 expression in Miz1ΔPOZ epidermis. Immunoblot of phosphorylated ERK (p-ERK), c-Myc and p53 in extracts of murine Control (Ctr) and Miz1ΔPOZ skin (A), untreated or treated with TPA (−/+TPA). α-tubulin was used as a loading control. The expression of p-ERK was also visualized in murine epidermis via immunohistochemistry in control (B, C) and Miz1ΔPOZ samples (D, E) both untreated (B, D) or TPA treated (C, E). Furthermore, p53 stained by immunohistochemistry in tumors did not reveal a difference between control (Ctr) and Miz1ΔPOZ papillomas (F, G), in contrast to p21cip1 expression (see Figure 4F, G and Figure S8). (TIF) [file pone.0034885.s005.tif]

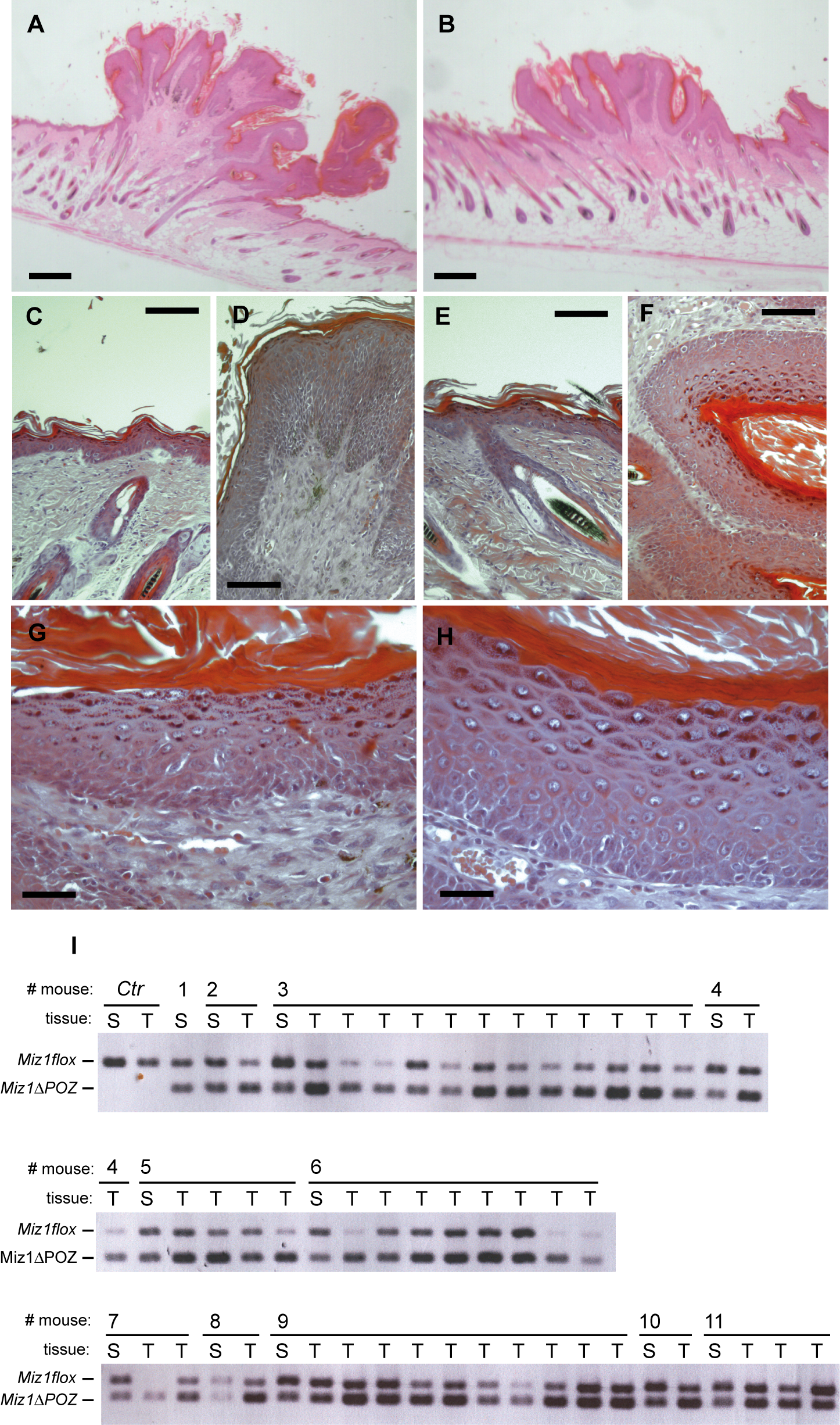

Supplement: Figure S6 — Histology and genotyping of Miz1ΔPOZ papillomas. HE-staining of control (A, C, D, G) and Miz1ΔPOZ papillomas (B, E, F, H). The length of the scale bars is 600 µm in A and B, 100 µm in C–F and 50 µm in G and H. (I) Miz1 genotyping of murine tail skin (S) and tumor tissue (T). A control (Ctr) animal and one tumor with a floxed Miz1 allele and no Cre recombinase expression was genotyped as a negative control. Animals 1–11 are Miz1ΔPOZ animals with a floxed Miz1 allele which express Cre recombinase. The lower band at 180 bp indicates the recombinant allele, while the upper band at 311 bp indicates the floxed allele. A floxed allele can also be detected in tumors from Miz1ΔPOZ animals due to the presence in the samples of other (non-keratinocyte) epidermal and dermal cell types that do not express Cre recombinase. (TIF) [file pone.0034885.s006.tif]

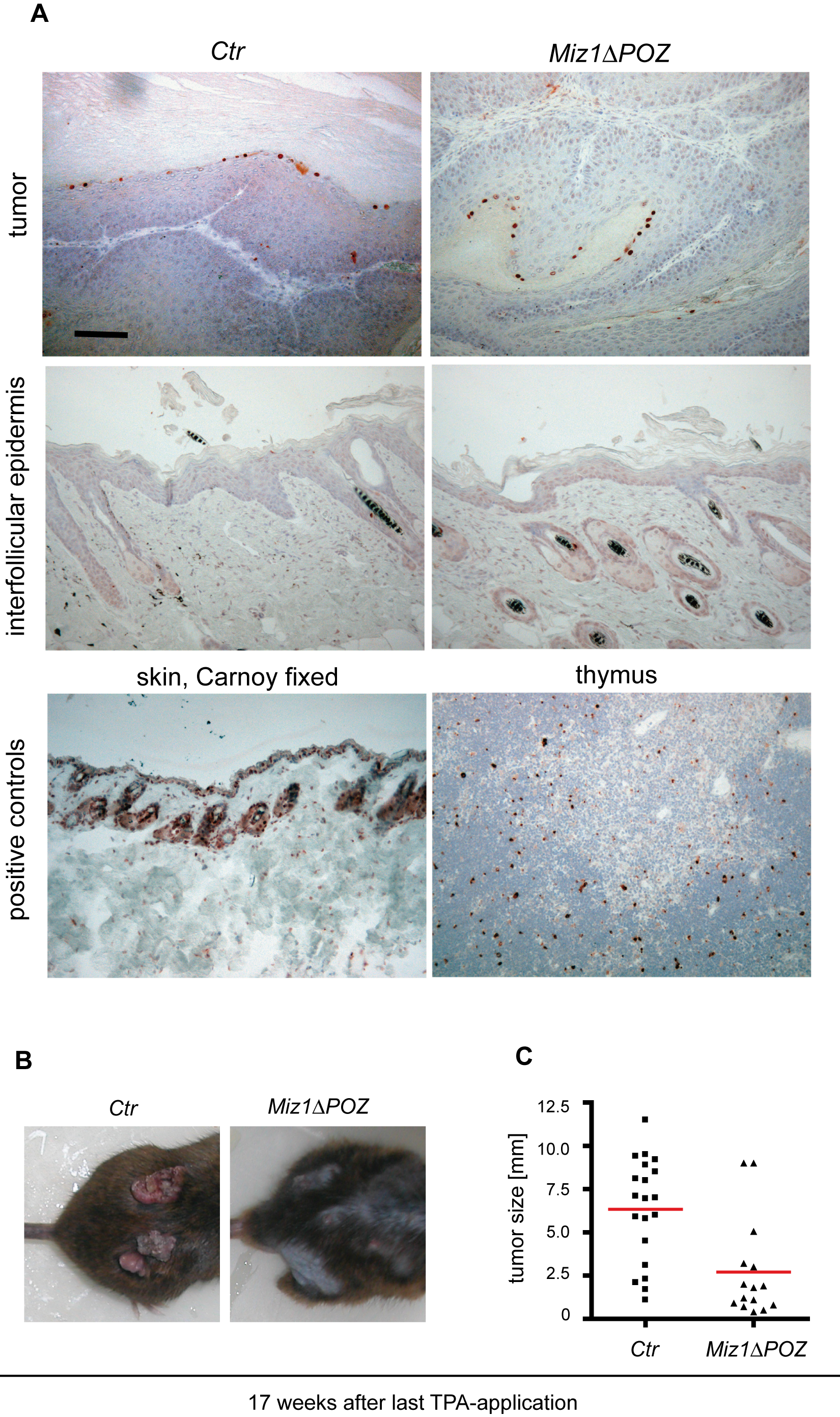

Supplement: Figure S7 — Apoptosis and tumor growth in tissue from control (ctr) and Miz1ΔPOZ animals. (A) While there were essentially no TUNEL positive cells in the interfollicular skin, we occasionally observed TUNEL positive cells in tumors independent of the genotype, although most tumors from both genotypes lacked TUNEL positive cells. As a positive control for the assay we used either skin fixed in Carnoy's solution, where most nuclei should be positive because of an acidic hydrolysis of the DNA (due to the acetic acid which is a component of this fixative) or thymus which usually exhibits a large number of apoptotic T-cells, predominately in the cortex. (B) Tumor development during 17 weeks after the last TPA treatment. Representative pictures of control (Ctr) and Miz1ΔPOZ papillomas 17 weeks after the last TPA treatment (B). Measurement of the tumor diameter (C) revealed an increased tumor-size in Ctr animals but not in Miz1ΔPOZ mice (compare with Figure 4H). (TIF) [file pone.0034885.s007.tif]

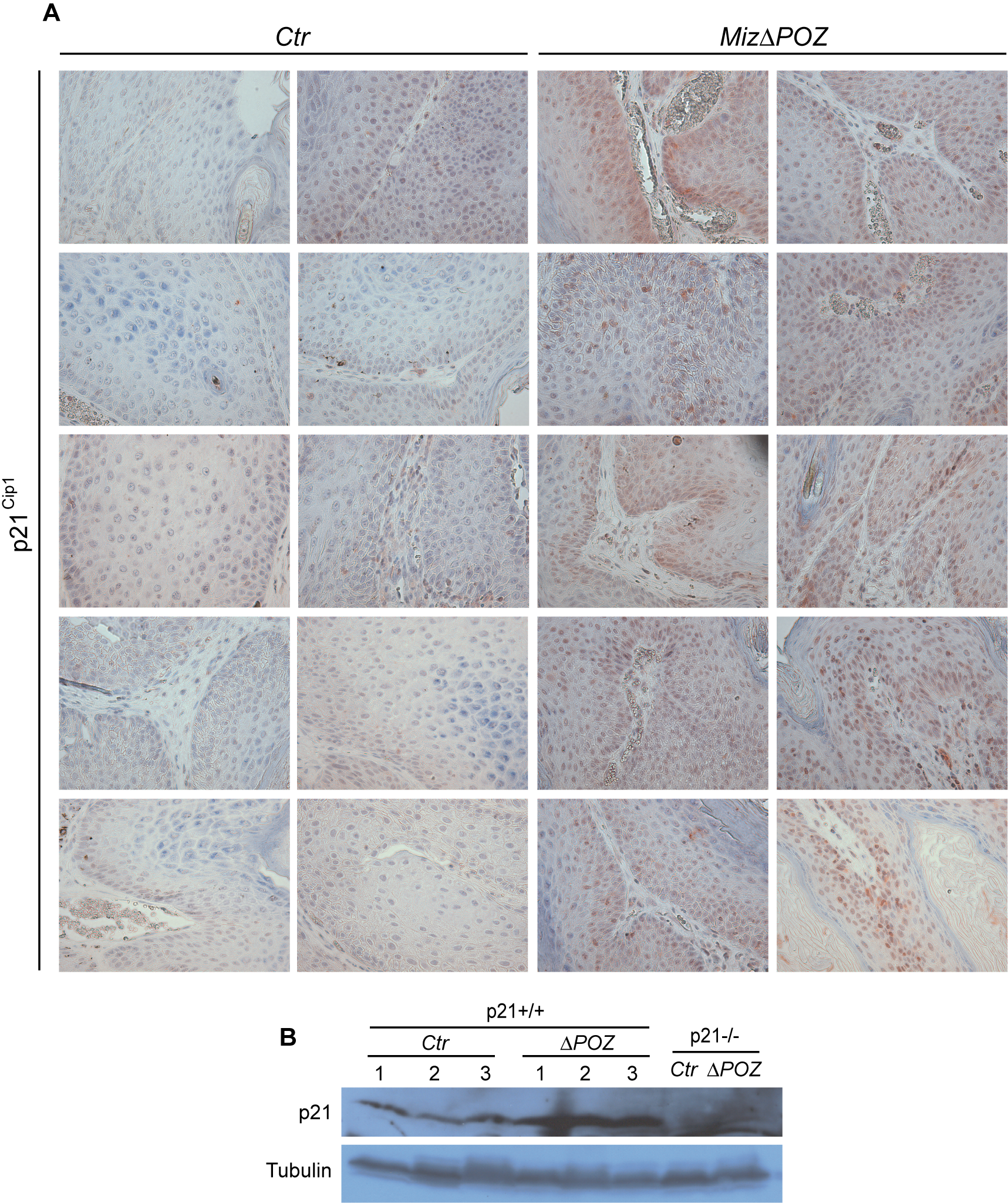

Supplement: Figure S8 — p21-expression in Miz1ΔPOZ papillomas. Immunohistochemistry of control (Ctr) and Miz1ΔPOZ papillomas showing the expression of p21cip1 (A). Each slide indicates a representative region of an individual tumor. In the majority of Miz1ΔPOZ papillomas, p21cip1 was upregulated, whereas in most Ctr tumors p21cip1 expression was not detectable. Expression of p21 protein in papillomas was also analyzed by immunoblot (B) in each of three (1–3) Ctr and Miz1ΔPOZ papillomas with p21+/+. Ctr and Miz1ΔPOZ tumor samples with a p21−/− background are negative controls. All Miz1ΔPOZ-tumors have an increased p21 expression compared to Ctr tumors, while in p21−/− tumors, no p21 expression was detectable. (TIF) [file pone.0034885.s008.tif]

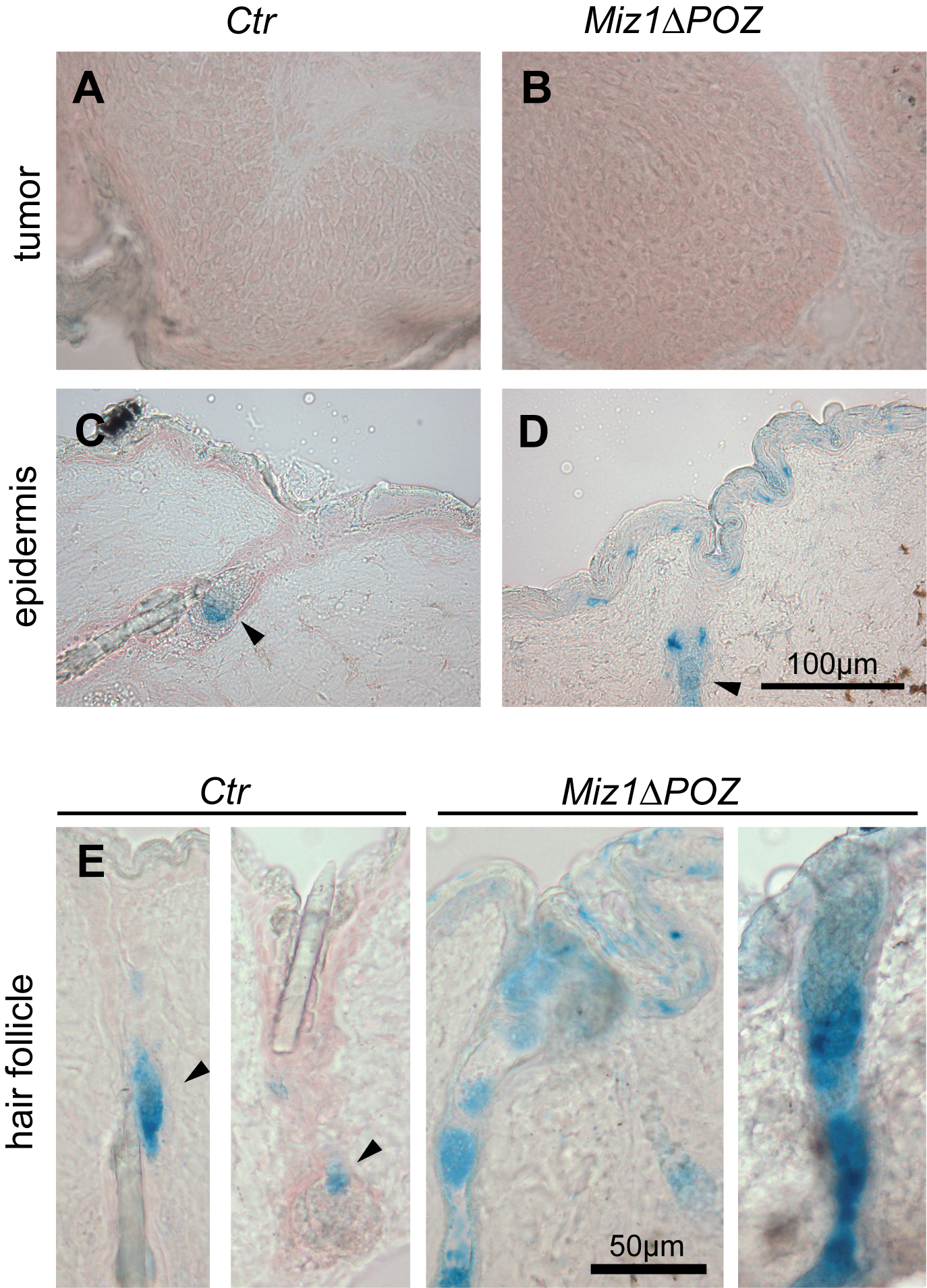

Supplement: Figure S9 — Tumors of Miz1ΔPOZ mice are not positive for SA-ß-galactosidase. Tumors from control (Ctr) (A) and Miz1ΔPOZ (B) animals after 20 weeks of TPA treatment were histochemically stained for SA-ß-galactosidase, but were not positive independent of the genotype. In contrast, skin from one year old Miz1ΔPOZ mice displayed a focal staining which was absent in Ctr animals (C, D). In addition, about 25% of hair follicles stained positive for SA-ß-galactosidase in Miz1ΔPOZ but not in Ctr animals (E). Arrowheads indicate sebaceous glands, which stain always positive for SA-ß-galactosidase. (TIF) [file pone.0034885.s009.tif]
